# Supplementary material for: DMRforPairs: identifying Differentially Methylated Regions between unique samples using array based methylation profiles
Source: BMC Bioinformatics. 2014 May 15;15:141. doi: 10.1186/1471-2105-15-141 (PMC4046028; doi:10.1186/1471-2105-15-141)
Supplement: Additional file 2 — DMRforPairs output for the comparison of A431-MCF7 and NA17018-NA17105. Please start from the HTML files in each folder. Available via the BMC Bioinformatics website. [file 1471-2105-15-141-S2.zip › 1394847754114233_MOESM2_ESM/A431_MCF7/figures/28201.pdf]

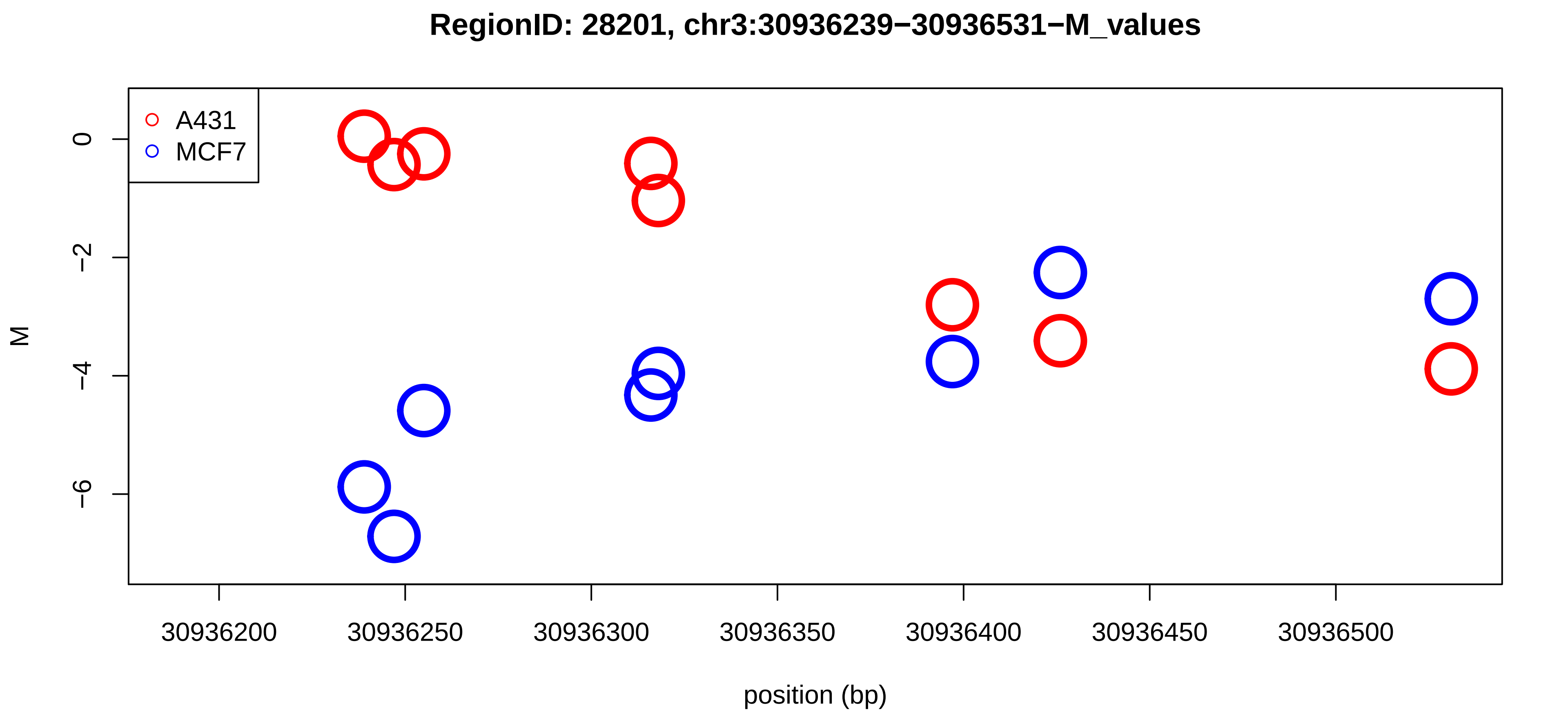

RegionID: 28201, chr3:30936239−30936531−M_values
A431
MCF7
M
0
−2
−4
−6
30936200
30936250
30936300
30936350
30936400
30936450
30936500
position (bp)

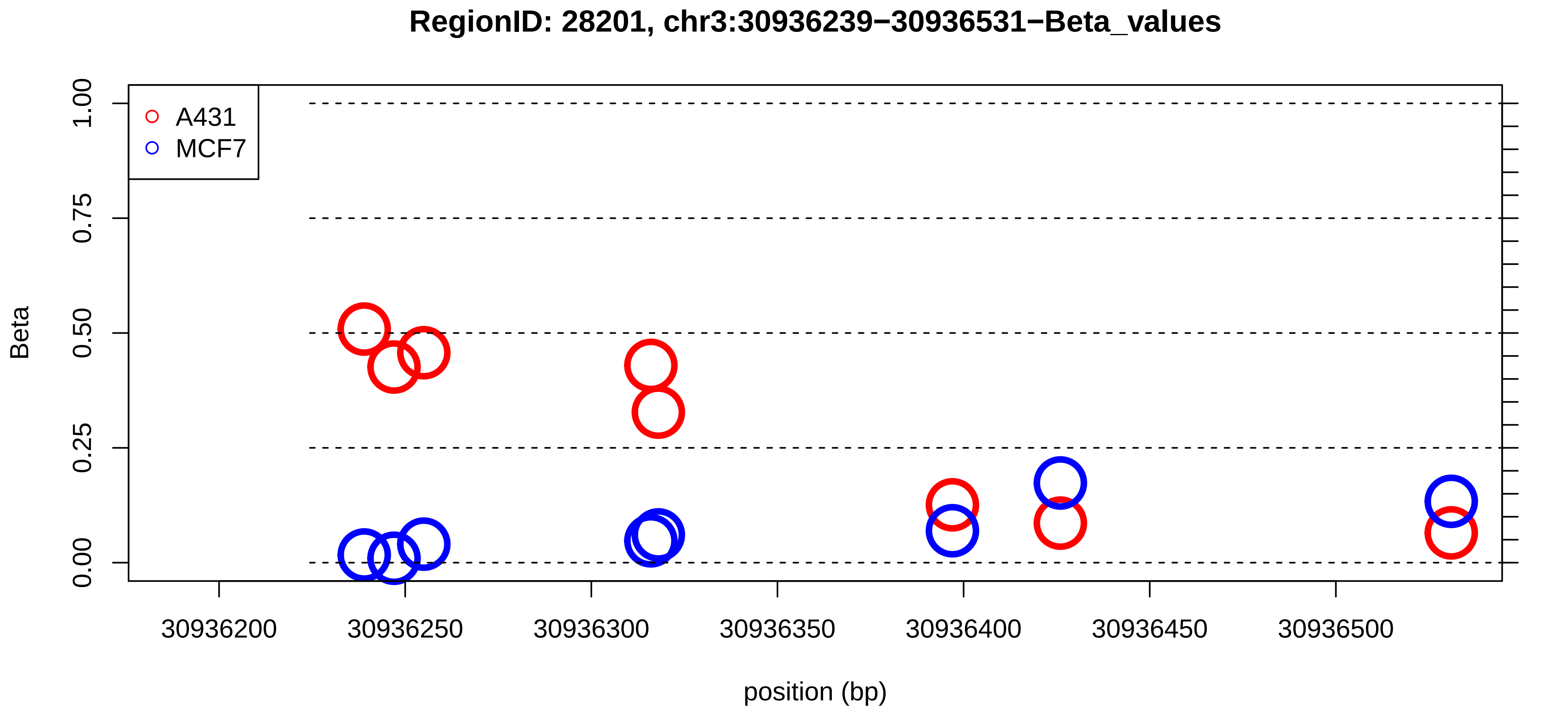

RegionID: 28201, chr3:30936239−30936531−Beta_values
A431
MCF7
Beta
0.00
0.25
0.50
0.75
1.00
30936200
30936250
30936300
30936350
30936400
30936450
30936500
position (bp)

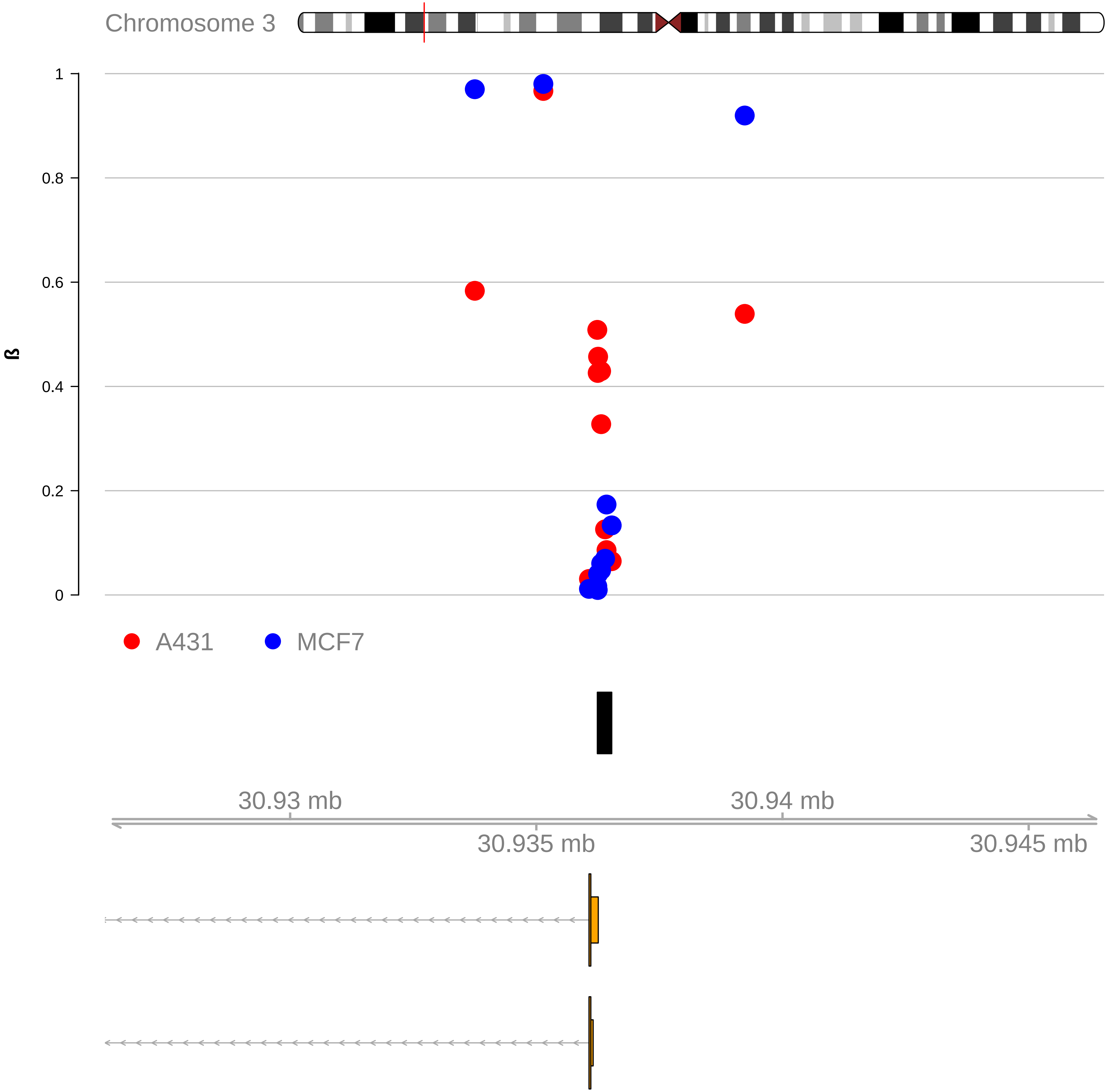

Chromosome 3
1
0.8
0.6
0.4
0.2
0
ß
A431
MCF7
30.93 mb
30.94 mb
30.935 mb
30.945 mb
